# DNA and mRNA Vaccines Against Hantaviruses: Antigen Design, Delivery Strategies and Translational Progress

**DOI:** 10.3390/molecules31193427

**Published:** 2026-09-26

**Authors:** Karolina Pawłuszkiewicz, Anita Maria Faltus, Martyna Zdych, Emilia Nowak, Emil Paluch

**Affiliations:** 1 Faculty of Medicine, Wroclaw Medical University, Wybrzeże L. Pasteura 1, 50-367 Wroclaw, Poland; karolina.pawluszkiewicz@student.umw.edu.pl (K.P.); anita.faltus@student.umw.edu.pl (A.M.F.); martyna.zdych@student.umw.edu.pl (M.Z.); 2 Department of Microbiology, Faculty of Medicine, Wroclaw Medical University, St. T. Chałubińskiego 4, 50-376 Wroclaw, Poland; apulapuczija@gmail.com

**Keywords:** hantavirus infections, Andes virus, orthohantaviruses, Puumala virus, vaccines, DNA, mRNA vaccines

## Abstract

Hantaviruses are members of the *Hantaviridae* family, which can cause hemorrhagic fever with renal syndrome (HFRS) and hantavirus cardiopulmonary syndrome (HCPS). At present, no specific antiviral treatment is available for hantavirus infection, while licensed hantavirus vaccines are restricted to a limited number of countries in Asia. This review summarizes recent advances in hantavirus deoxyribonucleic acid (DNA) and messenger RNA (mRNA) vaccines, focusing on antigen design, delivery systems, immunogenicity, safety and translational development. Peer-reviewed studies published primarily within the past 10 years were evaluated together with available hantavirus vaccine trials registered in ClinicalTrials.gov, with emphasis on Hantaan virus (HTNV), Puumala virus (PUUV) and Andes virus (ANDV). DNA vaccine candidates have demonstrated immunogenicity in preclinical and early clinical studies, with an acceptable safety profile. mRNA vaccines represent a newer approach with promising preclinical evidence of humoral and cellular immune responses. However, they have not yet been evaluated clinically. Studies have also reported the potential for varying degrees of cross-protection between hantavirus species, although protection remains limited by antigenic diversity. Overall, current evidence supports further development of nucleic acid-based hantavirus vaccines, particularly multivalent strategies, with emphasis on standardized evaluation and clinical efficacy.

## 1. Introduction

Hantaviruses are zoonotic viruses and members of the *Hantaviridae* family. They are maintained in nature by small mammalian reservoirs, particularly rodents and, in some cases, bats [1,2,3]. Each hantavirus genotype is usually linked to a specific rodent species. Populations of these animals fluctuate in response to ecological and environmental conditions such as food supply and climate conditions, with population expansions sometimes associated with increased human hantavirus cases [1,2,3,4]. Human infection occurs predominantly through the inhalation of aerosolized particles contaminated with excreta or secretions from infected rodents (via urine, saliva, and feces); in contrast, transmission through animal bites is uncommon [5]. Human-to-human transmission has been described for Andes virus (ANDV) [6,7,8], although its broader significance remains controversial [9]. Human hantavirus infections are generally classified into two major forms: hemorrhagic fever with renal syndrome (HFRS), including milder nephropathia epidemica caused by Puumala virus (PUUV), primarily linked to Old World hantaviruses in Europe and Asia; and hantavirus cardiopulmonary syndrome (HCPS), primarily linked to New World hantaviruses in the Americas [10,11,12]. Pathogenic Old World hantaviruses include, for example, Hantaan virus (HTNV), Seoul virus (SEOV), PUUV, Dobrava-Belgrade virus (DOBV) and Amur virus (AMV) [13,14], whereas New World hantaviruses include ANDV, Juquitiba virus (JUQV), Laguna Negra virus (LANV), Sin Nombre virus (SNV) and Araucaria virus (ARAV) [15,16,17].

The importance of hantaviruses has become increasingly apparent in the context of contemporary global events, including climate change, environmental disturbance, urban expansion, changes in agricultural practices, global travel and the growing recognition of zoonotic spillover as a major source of emerging infectious diseases. Environmental and climatic changes can influence the distribution, population density and ecological behavior of rodent reservoirs, thereby altering the frequency and geographical range of human exposure to hantaviruses [18]. At the same time, the COVID-19 pandemic has demonstrated the profound social, economic and healthcare consequences that can result from emerging viral pathogens and has substantially increased global awareness of the importance of pandemic preparedness and the rapid development of vaccine platforms. In this context, hantaviruses should be considered not only as established endemic pathogens but also as viruses of increasing strategic importance within the broader landscape of emerging zoonotic infections. The continuing risk of environmental change-driven shifts in reservoir ecology and the potential emergence of hantaviruses in previously unaffected regions further strengthen the rationale for developing flexible, rapidly adaptable vaccine platforms, including deoxyribonucleic acid (DNA) vaccines, capable of targeting high-priority pathogens such as HTNV [19].

At present, hantavirus vaccination remains an important but unresolved problem of disease prevention. Although inactivated vaccines have been used locally in China and the Republic of Korea, there is still no broadly available, internationally licensed vaccine for hantavirus infection in Europe, the United States or Latin America [18,19]. The World Health Organization (WHO) currently states that there is no licensed specific antiviral treatment or vaccine for hantavirus infection and clinical management remains primarily supportive, focused on early recognition and treatment of respiratory, cardiovascular and renal complications [19].

This review provides an updated overview of the most recent advances in hantavirus DNA and messenger ribonucleic acid (mRNA) vaccine development, with particular emphasis on the latest available clinical and preclinical studies. We focus on the evaluation of vaccine-induced immunogenicity, including neutralizing-antibody and cellular immune responses, as well as reported safety profiles and adverse events. In addition, we discuss the major methodological, immunological and translational challenges that currently limit further clinical development, including heterogeneity in vaccination schedules, delivery technologies and immunogenicity assays. Particular attention is given to the most extensively studied and clinically relevant hantaviruses, including HTNV, PUUV and ANDV.

## 2. Structure of Hantaviruses

Based on the International Committee on Taxonomy of Viruses (ICTV), hantaviruses are classified within the family *Hantaviridae* and the genus *Orthohantavirus*. They are enveloped RNA viruses with a negative polarity single-stranded genome with virions generally measuring approximately 80–100 nm in diameter [1,20]. As shown in Figure 1, the genome of hantaviruses consists of three RNA segments: the small (S) segment encodes the nucleocapsid (N) protein and in some hantaviruses a nonstructural protein (NSs), the medium (M) segment encodes a glycoprotein precursor (GPC), and the large (L) segment encodes the viral RNA-dependent RNA polymerase (RdRp) [21,22]. GPC is processed by the cellular signal peptidase complex at the conserved WAASA sequence, into the two viral envelope glycoproteins, Gn and Gc [23]. The nucleotide length of the S, M and L genome segments varies among hantavirus species and even between individual strains or isolates [24].

The viral genome is contained within ribonucleoprotein complexes (RNP), in which each RNA segment is encapsidated by the nucleoprotein (N) [25,26]. Hantavirus particles are surrounded by a host-derived lipid envelope originating from Golgi membranes. The lipid membrane is lined with 10 nm long glycoprotein spikes, composed of the viral envelope glycoproteins Gn and Gc [27]. Table 1 presents the hantavirus genome segments and the proteins they encode.

## 3. DNA Vaccines Against Hantaviruses

### 3.1. Mechanism of Action of DNA Vaccines

DNA vaccines are a nucleic acid vaccine platform in which plasmid DNA encoding a selected antigen is delivered into host cells [38,39]. Following administration, plasmid DNA must overcome several cellular barriers before antigen expression can occur. As a large, negatively charged molecule, DNA has limited ability to cross the plasma membrane and is therefore commonly delivered using viral vectors, physical methods such as electroporation or particle bombardment, or non-viral lipid- and polymer-based systems [40,41]. After cellular uptake, the plasmid must avoid intracellular degradation and reach the nucleus, where the encoded antigen gene is transcribed into mRNA. The mRNA is subsequently exported to the cytoplasm and translated into the antigen [40,41]. Thus, in contrast to mRNA vaccines, which can undergo translation following successful cytosolic delivery, DNA vaccines require successful nuclear translocation as an obligatory step for antigen production [38,39].

The efficiency of these delivery steps can influence the immunogenicity of DNA vaccines. Early approaches based on naked plasmid administration showed relatively low immunogenicity, attributed in part to limited plasmid uptake [41]. Delivery approaches such as electroporation can increase cellular uptake and transfection efficiency, while lipid- and polymer-based carriers can facilitate membrane transport and protect DNA from degradation [40,41]. In addition, optimizing the delivery route and targeting antigen-presenting cells can enhance antigen expression and shape the resulting immune response [41,42]. For hantavirus DNA vaccines, these factors may be particularly relevant because available experimental evidence suggests that both humoral and cellular immune responses can contribute to antiviral immunity [42]. Neutralizing antibodies against Gn and Gc can prevent viral entry into host cells. However, as hantaviruses are predominantly non-cytopathogenic, infected vascular endothelial cells can turn into active viral reservoirs. Cytotoxic T lymphocytes, particularly CD8+ T cells, may contribute to the recognition and elimination of infected cells. In addition, conservation of selected T-cell epitopes across hantavirus species may facilitate cross-reactive cellular immune responses, although the extent to which such responses translate into heterologous protection remains insufficiently established [5].

### 3.2. Antigen Design and Construction of DNA Vaccine Plasmids

The first HTNV DNA vaccine constructs were designed to express the two envelope glycoproteins, Gn and Gc (historically referred to as G1 and G2). These are the M-gene-based DNA vaccines. The viral M segment encodes a glycoprotein precursor [43]. Experimental studies have shown that it is processed in host cells and the resulting Gn and Gc glycoproteins are directed to the secretory pathway [27,43]. Mature Gn and Gc are major envelope antigens. Eventually, these can induce antibody responses, including virus-neutralizing antibodies [42,43,44,45,46,47,48].

The pWRG/HTN-M plasmid was generated using the pWRG7077 backbone, which contains regulatory elements required for antigen expression in mammalian cells, including the human cytomegalovirus immediate-early promoter, CMV intron A and the bovine growth hormone polyadenylation signal [27,43,45]. The HTNV M-segment coding sequence was inserted into this expression backbone and subsequently served as the basis for further optimization of HTNV DNA vaccine candidates [42,43]. Other bacterial backbone elements primarily support plasmid propagation and selection and are therefore not considered major determinants of antigen design.

Importantly, antigen design extends beyond optimization of the plasmid backbone and transcriptional elements. Structural characterization of hantavirus envelope glycoproteins has demonstrated that Gn and Gc form a highly organized surface lattice in which the Gc fusion protein adopts a metastable prefusion conformation [49]. Because conformational rearrangements of Gc are essential for membrane fusion, preservation of the prefusion state is an important consideration when designing recombinant glycoprotein immunogens. Structural studies of neutralizing antibodies have further shown that protective epitopes may involve multiple domains of Gc or span the Gn/Gc interface, indicating that antigenicity cannot always be predicted from linear sequence conservation alone [50,51].

Antigen selection should therefore consider both conserved and strain-specific epitopes, together with their structural accessibility and conformational integrity. In this context, antibody binding or cross-reactivity should be distinguished from neutralization and cross-neutralization, which represent functional inhibition of viral infection or entry. Protection represents a separate experimental endpoint demonstrated by reduced infection or disease following viral challenge and should not be equated with antibody binding, cross-neutralization, or clinical efficacy in humans [50,51,52].

These findings provide a structural rationale for the development of stabilized hantavirus glycoprotein immunogens. In particular, a recent HTNV study introduced structure-guided disulfide bonds to stabilize the glycoprotein in its prefusion conformation. The resulting GP-C3 construct elicited stronger neutralizing antibody responses than the corresponding non-stabilized constructs and was effective when delivered using both DNA and mRNA-LNP platforms. The enhanced response was associated with increased germinal-center activity and protection against high-dose HTNV challenge in mice [52]. Thus, structure-guided stabilization represents a promising complementary strategy to conventional nucleotide-level optimization of nucleic acid vaccine constructs.

Epitope selection may likewise benefit from structural characterization of antigenic sites. Structural studies of HTNV Gn have identified antibody-accessible regions capable of eliciting neutralizing responses, while studies of broadly neutralizing antibodies have identified conserved antigenic sites involving Gc and quaternary Gn/Gc epitopes [50,51,52]. Consequently, rational antigen design should consider not only sequence conservation but also epitope accessibility, structural conservation, conformational integrity and the ability of the engineered antigen to reproduce antigenic surfaces recognized by protective antibodies. Conserved epitopes may provide a basis for broader antibody recognition and neutralization across related viruses, whereas strain-specific epitopes may contribute predominantly to homologous responses. Therefore, the selection and structural presentation of antigenic regions can influence the breadth of the resulting neutralizing antibody response [53]. Integration of nucleotide sequence optimization with structure-guided protein engineering and rational epitope selection may therefore provide a more comprehensive framework for the development of next-generation hantavirus DNA and mRNA vaccines [53].

To refine the construct and eliminate non-essential sequences, a modified version was generated. The HTNV M-segment insert was PCR-amplified from pWRG/HTN-M using primer 1–24 together with the reverse primer (HTNMX, 5′-GCGCGGATCCGTTTGTGGTTAGAAAGCTAC). The product was cut with NotI and BamHI and ligated into the NotI-BglII-cut pWRG7077 vector. Although pWRG/HTN-M(x) retained the same HTNV coding region as the parental plasmid, it lacked part of the 3′ untranslated region and the vector sequence between BamHI and BglII [43], with approximately 100 undesirable nucleotides derived from the gene *nef* SIV [43]. Hooper et al. demonstrated that the deletion of these fragments did not result in a detectable reduction in expression of the cloned gene [43].

The final stage of construct optimization involved replacing the native viral sequence with a synthetically produced, codon-optimized M-segment open reading frame (ORF) and plasmid designated pWRG/HTN-M(co). This modification was intended to eliminate sequence features that could reduce transcript stability or limit efficient expression in mammalian cells. The optimized sequence, encoding the HTNV envelope glycoproteins Gn and Gc, was inserted into the pWRG7077 expression vector through the NotI and BglII restriction sites [43].

The similar strategy was also amplified to DNA vaccines targeting other pathogenic *Orthohantaviruses*. pWRG/PUU-M(s2), the PUUV DNA vaccine plasmid, contained a synthetic cDNA sequence based on a consensus of several PUUV isolates, which were optimized for codon usage (*Homo sapiens*) and mRNA stability [54]. In preclinical studies, it induced high-titer neutralizing antibodies in hamsters and nonhuman primates and protected vaccinated hamsters against PUUV infection. Protection was not observed against all related HFRS-associated hantaviruses. Interestingly, pWRG/PUU-M(s2) also protected hamsters against lethal ANDV disease despite no discernible ANDV cross-neutralizing antibodies [54]. The ANDV construct, pWRG/AND-M(opt2), was prepared by inserting the optimized M-segment ORF from the Chile-9717869 strain into the pWRG7077 vector, following the same general approach used for the HTNV and PUUV vaccines [55]. These constructs were first evaluated in preclinical models, for example, in rabbits and nonhuman primates. Vaccination induced high-titer neutralizing antibody responses [56].

These plasmid constructs were subsequently used as vaccine candidates in clinical studies [Table 2].

### 3.3. Immunogenicity of DNA Vaccines and Its Determinants

Immunogenicity is primarily assessed through the induction of neutralizing antibodies (NAbs), typically quantified using plaque reduction neutralization tests (PRNTs) [57,58]. The PRNT, originally developed by Dulbecco and colleagues in 1956, is a functional assay that evaluates the capacity of serum antibodies to inhibit viral entry and replication. Initially, this method was employed to monitor the evolution of dengue viruses by measuring virus-specific neutralizing antibody responses. Subsequently, PRNTs have been widely applied to correlate immune status with clinical outcomes, such as distinguishing asymptomatic from symptomatic dengue virus infections and evaluating vaccine-induced immunogenicity. At present, the PRNT is regarded as the gold standard for determining NAb titers in contexts where humoral immune responses are associated with protective immunity [58].

DNA vaccines encoding HTNV glycoproteins have consistently demonstrated the ability to elicit strong neutralizing antibody responses across multiple animal models, including hamsters, rabbits and nonhuman primates. Due to the absence of an ideal animal model that fully recapitulates human HFRS, preclinical evaluation has relied heavily on hamster infection models. In this system, vaccine efficacy is assessed indirectly by measuring infection following viral challenge [59]. In addition to inducing humoral immunity, these vaccines also stimulate cellular immune responses, notably CD4^+^ T-cell activation and cytokine production, which are essential for the establishment of effective and long-lasting immunity [57].

In vaccine studies, neutralizing antibodies targeting the envelope glycoproteins encoded by the M segment are quantified in sera from vaccinated hamsters using PRNT assays [60,61,62]. Following viral challenge, antibodies against the N protein are detected by enzyme-linked immunosorbent assay (ELISA) using recombinant N antigen produced in *Escherichia coli* [63]. Since the S genome segment encoding the N protein is not included in the vaccine construct, the absence of anti-N antibodies serves as an indicator of protection against infection with the challenge virus [64].

The pseudovirus neutralization assay (PsVNA) provides an alternative approach to the live-virus PRNT for measuring hantavirus-neutralizing antibodies, based on the same underlying principle but utilizing pseudotyped viral particles instead of live virus [65]. Ogino et al. [65] investigated vesicular stomatitis virus (VSV) pseudotypes (VSVG*), in which the G gene was replaced by the green fluorescent protein (GFP) gene, bearing HTNV or SEOV envelope glycoproteins Gn and Gc. The infectivity was evaluated in Vero E6 cells infected with VSVG*-HTN and -SEO. Both pseudotypes produced higher infectious titers than native HTNV and SEOV grown in Vero E6 cells. However, the measured neutralizing antibody titers were comparable between pseudotypes and native viruses. This neutralization system may be considered as an alternative to native hantaviruses, offering easier management because it requires less time to obtain results and no high-level containment laboratory, as the pseudotypes cannot produce infectious progeny in the absence of viral entry glycoproteins provided *in trans* [65].

Nevertheless, despite the practical advantages of pseudovirus-based assays and the generally strong correlation with conventional neutralization tests, the absolute neutralizing antibody titers obtained by PsVNA and PRNT should be interpreted with caution because the two assays measure partially different aspects of viral neutralization. PsVNA50 primarily measures the ability of antibodies to inhibit viral entry into target cells, whereas PRNT50 additionally reflects inhibition of viral egress and subsequent viral spread. These methodological differences may therefore contribute to discrepancies in the absolute titers obtained with the two assays, even when their results are strongly correlated [55]. This distinction was also demonstrated experimentally by Hooper et al. in a Phase 1 clinical trial of HTNV and PUUV DNA vaccines, in which PsVNA and PRNT results were directly compared [66]. The authors used Bland–Altman analyses to evaluate agreement and systematic differences between the two methods. For HTNV, PRNT50 values showed a negative bias relative to PsVNA50 values at all evaluated timepoints, indicating that the PRNT consistently yielded lower neutralizing antibody titers. In contrast, no systematic bias between PRNT50 and PsVNA50 was detected for PUUV [66]. Similar assay-dependent differences have also been observed for ANDV. Vial et al. [67] compared a single-round VSV-based PsVNA and a replication-competent recombinant VSV expressing ANDV glycoproteins neutralization assay with a focus reduction neutralization test (FRNT) using authentic ANDV. Although the VSV-based assays reproduced the general kinetics of neutralizing antibody responses observed with authentic virus, FRNT titers were consistently lower than those obtained with either VSV-based assay. The data indicate that neutralization titers generated using different assay platforms should not be considered directly interchangeable [67].

Importantly, no validated correlate of protection against hantavirus infection has yet been established in humans. Evidence from animal models nevertheless supports an association between neutralizing antibody titers and protection. In an experimental hamster model, passive transfer of antibodies derived from ANDV DNA-vaccinated macaques was associated with complete protection against lethal ANDV challenge at recipient PRNT50 titers of 320–1280. However, these values were obtained in an animal passive-transfer model and should not be extrapolated directly to humans or interpreted as established neutralizing antibody thresholds in clinical settings [43,55].

The delivery of the DNA vaccines can significantly influence immunogenicity. Previous studies have focused primarily on gene gun and intramuscular (IM) electroporation, whereas more recent studies have explored simpler delivery methods by using disposable syringe and needle-free injection [56,68,69,70]. Physical delivery methods, particularly electroporation, can enhance the immunogenicity of DNA vaccines compared with conventional needle-and-syringe administration. This conventional administration, done by needle and syringe, leads to the deposition of DNA in tissues [56,66,69].

Other delivery methods were developed to facilitate the administration [56,68,69]. The TriGrid Delivery System combines the delivery with electroporation, the method increasing the cellular uptake of plasmid DNA [66,69,70,71]. Upon DNA injection, the electrical pulses are applied, causing the transient disruption and permeability of cell membranes [66].

An alternative method, which uses high-pressure fluid jet to deposit DNA in tissue, is PharmaJet Stratis^®^ Needle-Free Jet Injection [55,66]. Unlike the TriGrid System, it does not use electroporation, is operationally simpler and does not require an electroporation pulse generator or electrode array. This method was used, for example, in a Phase I clinical trial of HTNV and PUUV DNA vaccines [66].

DNA vaccine immunogenicity is influenced not only by the delivery technology but also by the route of administration, dose, vaccine construct and vaccination schedule [51,60]. Kwilas et al. [56] in a preclinical study on immunogenicity of an SNV DNA vaccine were comparing the intramuscular (IM) and intradermal (ID) route using the Pharmajet Stratis^®^ System. In rabbits, the seroconversion after the first dose reached 100% (4/4) in both the 0.4 mg IM group and 0.4 mg ID group; after the third dose, seroconversion remained at 100% in both groups. In rhesus macaques that received 1.0 mg IM, seroconversion was 100% (3/3) after the first dose and 100% after the third dose. However, in rhesus macaques receiving 1.0 mg ID, only 1/3 animals seroconverted after the first dose, but 3/3 animals reached 100% (*p*-value 0.0115) seroconversion after the third dose. It is important to note the presence of species-specific differences in skin structure, which is a significant factor for ID route. Another consideration is injection volume of the liquid vaccine solution—the ID route required a smaller volume than the IM route [56].

Although it is not possible to directly compare the results of both methods, needle-free jet injection (PharmaJet Stratis) and electroporation-based delivery (TriGrid) both resulted in inducing neutralizing antibody responses in clinical trials [66,69,70,71]. The important element is not only the efficiency of DNA delivery, but also the reproducibility and practicality of the vaccination procedure [66]. Electroporation requires the application of electrical impulses and another device, while needle-free jet injection does not use electroporation and eliminates the conventional needle [66]. The progression from using the gene gun method to IM electroporation and needle-free injection reflects efforts to make simpler, more practical hantavirus DNA vaccine procedures, all while maintaining immunogenicity [66,69,70].

Preclinical studies have demonstrated that DNA vaccination against HTNV confers protective immunity not only against homologous HTNV challenge but also against related hantaviruses, including SEOV and DOBV, indicating the presence of cross-protective immune responses. However, protection against more antigenically divergent viruses, such as PUUV, remains limited, thereby necessitating the development of multivalent vaccine formulations to achieve broader coverage [43,52,57,59].

Despite these promising findings, several challenges have emerged in preclinical evaluations. A major limitation is the occurrence of immune interference in multivalent vaccine formulations. Specifically, when HTNV and PUUV DNA vaccines are co-administered, the immune response to HTNV is frequently diminished, whereas responses to PUUV predominate. This phenomenon presents a significant obstacle to the development of broadly protective hantavirus vaccines [57].

In addition to safety assessments, serum neutralizing antibody responses to HTNV and PUUV were evaluated in samples collected on days 0 and 57 from a cohort of eighty rabbits. PRNT analysis of day 57 sera demonstrated that 100% (*p*-value < 0.0001) of rabbits vaccinated with the HTNV DNA vaccine, PUUV DNA vaccine, or a combination of both developed neutralizing antibodies against HTNV and/or PUUV following a three-dose immunization regimen. Neutralizing antibody titers against HTNV in sera from rabbits vaccinated with either the HTNV DNA vaccine alone or the combined HTNV and PUUV vaccine ranged from <20 to 40,960. Similarly, PUUV-specific neutralizing antibody titers in sera from rabbits vaccinated with the PUUV DNA vaccine alone or in combination ranged from 40 to 40,960. The homotypic PRNT_50_ geometric mean titers (GMTs) for the PUUV and HTNV vaccine groups were 2388 and 7828, respectively. Meaning, that both vaccines were highly effective at producing neutralizing antibodies in rabbits when given on their own. The HTNV vaccine produced a higher average level of antibodies than the PUUV vaccine, but both results were considered strong indicators that the vaccines were “biologically active and immunogenic” [64].

To address the issue of vaccine interference observed in hamsters, rabbits and humans, modified vaccine ratios were evaluated in hamster models. Animals were immunized three times at approximately three-week intervals via IM-electroporation (IM-EP) with a total dose of 100 μg DNA, administered either as individual vaccines or as mixtures. Tested HTNV:PUUV DNA ratios included 1:1, 2:1 and 10:1. Consistent with previous observations, the 1:1 vaccine mixture elicited neutralizing antibodies against PUUV but not HTNV. Increasing the proportion of HTNV DNA to 2:1 or 10:1 did not alter this outcome, as no detectable HTNV-specific neutralizing antibody response was observed by PRNT [64].

Recent advances in antigen design, particularly prefusion stabilization of glycoproteins, represent a significant step forward in improving vaccine efficacy. These structure-based approaches enhance the presentation of neutralizing epitopes and promote stronger and more durable immune responses. Furthermore, strategies such as codon optimization, synthetic gene design and prime–boost regimens are being explored to further enhance immunogenicity [52,72]. Further optimization of antigen design and delivery methods will be required to improve the consistency and magnitude of vaccine-induced immune responses.

### 3.4. Clinical Evaluation of Hantavirus DNA Vaccines

DNA vaccines targeting pathogenic *orthohantaviruses* have progressed into clinical evaluation with efforts concentrated primarily on HTNV and PUUV, and, more recently, on ANDV. Table 2, Table 3 and Table 4 summarize clinical trials of DNA vaccines against HTNV, PUUV and ANDV registered in ClinicalTrials.gov from 2013 to 2026. The data were searched on 4 August 2026 using the following terms: “Puumala” AND “vaccine”, “Hantaan” AND “vaccine”, “Andes” AND “vaccine”, as well as the corresponding abbreviations: “PUUV” AND “vaccine”, “HTNV” AND “vaccine”, and “ANDV” AND “vaccine”. Six clinical trials were identified: five evaluated monovalent or combined DNA vaccines against HTNV and PUUV, whereas one evaluated a DNA vaccine against ANDV.

**Table 2:** General characteristics of clinical trials evaluating DNA vaccines against HTNV, PUUV and ANDV.

Vaccine Target	Trial ID, Phase and Status	Last Posted Update	Sponsor	Number of Enrolled and Analyzed Participants	Dose and Vaccination Schedule	Delivery Method	Name of Vaccine Plasmid	References
HTNV DNA vaccine, PUUV DNA vaccine, mixed HTNV/PUUV vaccine	NCT01502345, phase I, completed	31 January 2013	U.S. Army Medical Research and Development Command	31 enrolled; 27 completed study procedures	Group 1: HTNV DNA vaccine, 2 mg, 3-dose regimen—Days 0, 28 and 56 Group 2: PUUV DNA vaccine, 2 mg, 3-dose regimen—Days 0, 28 and 56 Group 3: combined HTNV/PUUV DNA vaccine, 2 mg total (1 mg HTNV + 1 mg PUUV), three-dose regimen—Days 0, 28 and 56.	IM administration using the TriGrid Delivery System	pWRG/HTN-M(x); pWRG/PUUV-M(s2)	[73]
Mixed HTNV and PUUV DNA vaccine	NCT02116205, phase IIa, completed	12 February 2021	U.S. Army Medical Research and Development Command	130 enrolled; 120 completed study procedure	Cohort 1: 2 mg, four-dose regimen—Days 0, 28, 56, 168 Cohort 2: 2 mg, three-dose regimen—vaccine on Days 0, 56, 168; placebo on Day 28 Cohort 3: 1 mg, four-dose regimen—Days 0, 28, 56, 168 Cohort 4: 1 mg, three-dose regimen—vaccine on Days 0, 56, 168; placebo on Day 28	IM administration by electroporation using the TriGrid Delivery System	pWRG/HTN-M(co); pWRG/PUU-M(s2)	[74]
HTNV DNA vaccine, PUUV DNA vaccine, mixed HTNV/PUUV DNA vaccines	NCT02776761, phase I, completed	16 February 2021	U.S. Army Medical Research and Development Command	27 enrolled; 22 completed study procedure	Group 1: HTNV DNA vaccine, 2 mg/vaccination; Group 2: PUUV DNA vaccine, 2 mg/vaccination; Group 3: combined HTNV/PUUV DNA vaccine, 2 mg/vaccination total	IM administration using the Pharmajet Stratis^®^ Needle-Free Jet Injection Delivery Device	pWRG/HTN-M(co); pWRG/PUU-M(s2)	[75]
HTNV vaccine, PUUV vaccine	NCT03718130, phase I, Completed	26 June 2025	U.S. Army Medical Research and Development Command	61 enrolled, 35 completed study procedure by day 220	0.6 mg ID or 3.0 mg for monovalent HTNV or PUUV vaccine; 1.2 mg ID or 6.0 mg IM for the combined HTNV/PUUV vaccine, administered on Days 0, 28 and 56	IM or ID administration using the TriGrid Delivery System	pWRG/HTN-M(co); pWRG/PUU-M(s2) (data from clinical trial form)	[71]
HTNV vaccine, PUUV vaccine	NCT04333459, phase II, Completed	19 May 2026	U.S. Army Medical Research and Development Command	132 enrolled	1 or 2 mg of HTNV or PUUV DNA on Days 1, 29, 57 and 169	IM administration using the Pharmajet Stratis^®^ Needle-Free Jet Injection Delivery Device	pWRG/HTN-M(co); pWRG/PUU-M(s2)	[76]
ANDV vaccine	NCT03682107, phase I, Completed	22 November 2022	NIAID	48 enrolled; 48 completed study procedure	Cohort 1: ANDV DNA 2 mg, three-dose regimen—Days 1, 29, 169 Cohort 2: ANDV DNA 2 mg, four-dose regimen—Days 1, 29, 57, 169 Cohort 3: ANDV DNA 4 mg, three-dose regimen—Days 1, 29, 169 Cohort 4: ANDV DNA 4 mg, four-dose regimen—Days 1, 29, 57, 169	IM administration using the Pharmajet Stratis^®^ Needle-Free Jet Injection Delivery Device	pWRG/AND-M (opt2)	[77]

Abbreviations: HTNV—Hantaan virus; PUUV—Puumala virus; ANDV—Andes virus; DNA—deoxyribonucleic acid; IM—intramuscular; ID—intradermal; NIAID—National Institute of Allergy and Infectious Diseases; U.S.—United States; mg—milligram.

**Table 3:** Summary of local and systemic adverse events (AEs) of DNA vaccines against HTNV, PUUV and ANDV tested in clinical trials.

Vaccine Target	Trial ID	Local AEs	Systemic AEs	SAEs in Clinical Trial	References
HTNV DNA vaccine, PUUV DNA vaccine, mixed HTNV/PUUV vaccine	NCT01502345	Injection-site pain: 28/31 (90.3%; 95% CI 74.2–98.0); bruising: 7/31 (22.6%; 95% CI 9.6–41.1); erythema: 3/31 (9.7%; 95% CI 2.0–25.8)	Headache: 8/31 (25.8%; 95% CI 11.9–44.6); myalgia: 7/31 (22.6%; 95% CI 9.6–41.1); fatigue: 6/31 (19.4%; 95% CI 7.5–37.5); muscle contractions and tachypnea: two participants each	No SAEs related to the vaccine or study-related procedures were observed. One unrelated Grade 4 hypoglycemia case was attributed to a previously unrecognized condition.	[69,73]
Mixed HTNV and PUUV DNA vaccine	NCT02116205	Injection-site pain: 125/130 (96.2%; 95% CI 91.3–98.7); erythema: 18/130 (13.8%; 95% CI 8.4–21.0); bruising: 9/130 (6.9%; 95% CI 3.2–12.7); swelling: 4/130 (3.1%; 95% CI 0.8–7.7)	Fatigue: 38/130 (29.2%; 95% CI 21.6–37.8); headache: 31/130 (23.8%; 95% CI 16.8–32.1); myalgia: 23/130 (17.7%; 95% CI 11.6–25.4)	No SAEs related to the vaccine or study-related procedures were observed.	[70,74]
HTNV DNA vaccine, PUUV DNA vaccine, mixed HTNV/PUUV DNA vaccines	NCT02776761	Injection-site pain: 21/27 (77.8%; 95% CI 57.7–91.4); bruising: 11/27 (40.7%; 95% CI 22.4–61.2); erythema: 8/27 (29.6%; 95% CI 13.8–50.2)	Headache: 13/27 (48.1%; 95% CI 28.7–68.1); fatigue: 10/27 (37.0%; 95% CI 19.4–57.6); ≥1 solicited related AE: 23/27 (85.2%; 95% CI 66.3–95.8)	No SAEs related to the vaccine or study-related procedures were observed.	[66,75]
ANDV vaccine	NCT03682107	≥1 local solicited AE: 47/48 (97.9%; 95% CI 88.9–99.9); erythema: 43/48 (89.6%; 95% CI 77.3–96.5); induration: 43/48 (89.6%; 95% CI 77.3–96.5); tenderness: 39/48 (81.3%; 95% CI 67.4–91.1); pain: 32/48 (66.7%; 95% CI 51.6–79.6); bruising: 20/48 (41.7%; 95% CI 27.6–56.8); skin discoloration: 13/48 (27.1%; 95% CI 15.3–41.8)	≥1 systemic solicited AE, vaccine: 28/40 (70.0%; 95% CI 53.5–83.4); placebo: 3/8 (37.5%; 95% CI 8.5–75.5); mild fever: 3/40 (7.5%; 95% CI 1.6–20.4)	No SAEs related to the vaccine or study-related procedures were observed. One SAE—acute rhabdomyolysis—occurred in 1/48 participants and was considered unrelated to vaccination.	[55,77]

Abbreviations: HTNV—Hantaan virus; PUUV—Puumala virus; ANDV—Andes virus; DNA—deoxyribonucleic acid; AE—adverse event; SAEs—serious adverse events; CI—Confidence Interval. Percentages are presented together with the corresponding numbers of participants (*n/N*). Where the numerator and denominator were available, 95% CIs for proportions were calculated using the exact Clopper–Pearson method.

**Table 4:** Summary of serological response and neutralizing antibody titers of DNA vaccines against HTNV, PUUV and ANDV tested in clinical trials.

Vaccine Target	Trial ID	Study Group and Number of Participants	Immunogenicity Assay	Definition of Response	Immunogenicity Outcomes	References
HTNV DNA vaccine, PUUV DNA vaccine, mixed HTNV/PUUV vaccine	NCT01502345	*n* = 31 healthy adults; HTNV: *n* = 11 (≥2 doses; 9 completed three doses), PUUV: *n* = 9, HTNV + PUUV: *n* = 9	PRNT_50_	A detectable response was defined as a PRNT_50_ titer ≥20.	HTNV: 7/11 (63.6%; 95% CI 30.8–89.1); PUUV: 6/8 (75.0%; 95% CI 34.9–96.8); combined vaccine: PUUV 7/9 (77.8%; 95% CI 40.0–97.2); both viruses: 3/9 (33.3%; 95% CI 7.5–70.1) after ≥2 doses.	[69,73]
Mixed HTNV and PUUV DNA vaccine	NCT02116205	*n* = 130 healthy adults; Cohort 1: *n* = 30, 2 mg HTNV + PUUV DNA, four vaccine doses; Cohort 2: *n* = 30, 2 mg HTNV + PUUV DNA, three vaccine doses; Cohort 3: *n* = 30, 1 mg HTNV + PUUV DNA, four vaccine doses; Cohort 4: *n* = 30, 1 mg HTNV + PUUV DNA, three vaccine doses; 10 additional participants were enrolled as alternates.	PsVNA_50_ and PRNT_50_ on Day 84	Seropositivity was generally defined as a neutralizing-antibody titer ≥20.	HTNV: Cohort 1 24/30 (80.0%; 95% CI 61.4–92.3); Cohort 2 27/30 (90.0%; 95% CI 73.5–97.9); Cohort 3 27/30 (90.0%; 95% CI 73.5–97.9); Cohort 4 25/30 (83.3%; 95% CI 65.3–94.4). PUUV: Cohort 1 26/30 (86.7%; 95% CI 69.3–96.2); Cohort 2 24/30 (80.0%; 95% CI 61.4–92.3); Cohort 3 25/30 (83.3%; 95% CI 65.3–94.4); Cohort 4 22/30 (73.3%; 95% CI 54.1–87.7). Response to both viruses: Cohort 1 23/30 (76.7%; 95% CI 57.7–90.1); Cohort 2 22/30 (73.3%; 95% CI 54.1–87.7); Cohort 3 24/30 (80.0%; 95% CI 61.4–92.3); Cohort 4 20/30 (66.7%; 95% CI 47.2–82.7).	[70,74]
HTNV DNA vaccine, PUUV DNA vaccine, mixed HTNV/PUUV DNA vaccines	NCT02776761	*n* = 27 healthy adults; Cohort 1: *n* = 9, HTNV DNA vaccine; Cohort 2: *n* = 9, PUUV DNA vaccine; Cohort 3: *n* = 9, combined HTNV + PUUV DNA vaccine. Immunogenicity population: *n* = 22 (7, 6 and 9, respectively).	PsVNA_50_ and PRNT_50_	Seropositivity was defined as a titer ≥20, whereas seroconversion in initially seronegative participants was defined as a titer ≥40.	PsVNA_50_ seroconversion: HTNV 7/7 (100%; 95% CI 59.0–100); PUUV 6/6 (100%; 95% CI 54.1–100). Combined cohort: PUUV-neutralizing antibodies 8/9 (88.9%; 95% CI 51.8–99.7); HTNV-neutralizing antibodies 7/9 (77.8%; 95% CI 40.0–97.2); both viruses 7/9 (77.8%; 95% CI 40.0–97.2). By PRNT_50_, homologous seroconversion: HTNV 7/7 (100%; 95% CI 59.0–100); PUUV 5/6 (83.3%; 95% CI 35.9–99.6); combined group, both viruses seropositive 4/9 (44.4%; 95% CI 13.7–78.8); titers ≥40 against both viruses 3/9 (33.3%; 95% CI 7.5–70.1).	[66,75]
ANDV vaccine	NCT03682107	*n* = 48 healthy adults; Cohort 1: *n* = 10, 2 mg ANDV DNA, three-dose schedule; Cohort 2: *n* = 10, 2 mg, four-dose schedule; Cohort 3: *n* = 10, 4 mg, three-dose schedule; Cohort 4: *n* = 10, 4 mg, four-dose schedule; Placebo: *n* = 8.	PsVNA_50_ as the principal immunogenicity assay and confirmed using PRNT_50_	Seropositivity (titer ≥20) and seroconversion (≥40 if the baseline was <20, or a minimum 4-fold rise compared to baseline if the baseline titer was ≥20)	Cohorts 2–4 achieved at least 80% seropositivity by Day 197, which was sustained through Day 337. At the final assessment, seropositivity was 67% (66.7%; 95% CI 29.9–92.5) in Cohort 1 and approximately 88–90% in Cohorts 2–4. The highest reported final seroconversion frequencies were 88% in Cohort 2 and 89% in Cohort 4.	[55,77]

Abbreviations: HTNV—Hantaan virus; PUUV—Puumala virus; DNA—deoxyribonucleic acid; PRNT_50_—50% plaque reduction neutralization test; PsVNA_50_—50% pseudovirion neutralization assay; mg—milligram; CI—Confidence Interval. Serological response rates are presented as *n*/*N*, percentage and 95% CI where the underlying numerator and denominator were available. The 95% CIs for proportions were calculated using the exact Clopper–Pearson method.

Table 3 summarizes the local and systemic adverse events (AEs) reported in the registered clinical trials evaluating DNA vaccines against HTNV, PUUV and ANDV. Clinical trials were excluded from the quantitative comparison when their results had not been reported in a peer-reviewed scientific publication. Accordingly, NCT03718130 was excluded because the available findings were limited to the ClinicalTrials.gov registry and had not been published in a peer-reviewed journal [71]. NCT04333459 was excluded because no study results had been publicly posted or published at the time of the literature search [76].

Because most of the studies were phase I or early phase II trials, safety and tolerability were the most analyzed elements. Overall, the evaluated hantavirus DNA vaccines shown in Table 3 have an acceptable safety profile. Injection-site pain was the most frequently reported local reaction, occurring in 77.8–96.2% of participants in the HTNV/PUUV studies. Other common local events included erythema, bruising, swelling, induration, tenderness and skin discoloration. The high frequency of local reactions in studies using electroporation or needle-free jet injection suggests that many of these reactions may be related not only to the plasmid vaccine itself but to the delivery method.

The most frequently reported systemic AEs were headache, fatigue, myalgia and malaise. In the HTNV/PUUV trials, these reactions were generally mild or moderate and self-limiting.

No vaccine- or procedure-related serious adverse events (SAEs) were reported in these studies. Several unrelated SAEs, including hypoglycemia and acute rhabdomyolysis, were recorded in individual trials but were not considered causally related to vaccination [55,69,73,77].

Table 4 summarizes the available clinical data on the humoral immune response elicited by DNA vaccines against HTNV, PUUV and ANDV, including seroconversion rates and neutralizing antibody titers.

To provide a quantitative synthesis while accounting for the substantial methodological heterogeneity across clinical studies, response rates were evaluated at the individual-study level together with their corresponding 95% confidence intervals. The Phase 1 study (NCT01502345) demonstrated neutralizing-antibody responses in 7/11 participants receiving the HTNV DNA vaccine (63.6%; 95% CI, 30.8–89.1%) and 6/8 participants receiving the PUUV DNA vaccine (75.0%; 95% CI, 34.9–96.8%). In the combined HTNV/PUUV group, 7/9 participants (77.8%; 95% CI, 40.0–97.2%) developed PUUV-neutralizing antibodies, whereas 3/9 (33.3%; 95% CI, 7.5–70.1%) responded to both viruses. These estimates illustrate the considerable uncertainty associated with the small sample sizes of early-phase trials [69,73].

The available clinical data indicate that *orthohantavirus* DNA vaccines are capable of inducing measurable neutralizing-antibody responses against HTNV, PUUV and ANDV. The earliest HTNV/PUUV study, NCT01502345, demonstrated proof-of-concept immunogenicity, but response rates were moderate and heterogeneous, whereas the combined formulation produced a markedly stronger response to PUUV than to HTNV. This asymmetry suggests that simply combining two plasmids does not necessarily preserve the immunogenicity of each component and raises the possibility of antigenic or immunological interference within bivalent formulations [73].

Later studies demonstrated that repeated dosing and booster administration could increase both the proportion of responders and neutralizing-antibody titers, suggesting that vaccination schedule is an important determinant of immunogenicity and may be at least as relevant as the absolute plasmid dose [55,66,69,70,73,74,75,77].

A notable limitation of the current evidence is the lack of methodological uniformity across trials. Different studies used distinct plasmid constructs, dosing regimens, delivery devices, routes of administration and sampling schedules. Neutralizing antibody titers obtained using different assay platforms, including PRNT and PsVNA, should not be directly compared because these methods differ in their biological readouts, analytical sensitivity and assay conditions [66,67]. Moreover, no validated correlate of protection has yet been established for hantavirus vaccines in humans; therefore, the reported neutralizing antibody titers should be interpreted as measures of immunogenicity rather than as defined thresholds of clinical protection [55]. However, evidence from experimental models indicates that protection may also occur in the absence of detectable neutralizing antibodies, suggesting that cellular immune responses may contribute to protection [57,64]. Consequently, the extent to which neutralizing antibodies alone, cellular immunity, or their combination determines protection in humans remains uncertain and no validated immune threshold can currently be used to define protection following hantavirus vaccination. In addition, seropositivity and seroconversion were not defined consistently across the clinical development program [55,66,69,70,73,74,75,77]. Importantly, the available clinical evidence remains limited to early-phase studies primarily designed to assess safety and immunogenicity rather than protective clinical efficacy. Subsequent Phase 2a evaluation included 120 participants but primarily examined the effects of vaccine dose and administration schedule on neutralizing-antibody responses, with four vaccination cohorts differing in dose and whether DNA vaccine or placebo was administered on Day 28 [70]. Similarly, the more recent Phase 1 study using needle-free injection evaluated safety and immunogenicity in small cohorts and employed different vaccine formulations and vaccination schedules [66]. Thus, although these studies demonstrate that DNA vaccination can induce neutralizing antibodies, they do not demonstrate protective clinical efficacy against hantavirus infection or disease.

Taken together, the available clinical data support the continued development of DNA vaccines against *orthohantaviruses*, particularly with further optimization of multidose vaccination schedules. The results obtained so far indicate that these vaccines can induce neutralizing antibody responses and generally show an acceptable safety profile, although the magnitude and consistency of the immune response vary between studies and vaccination regimens. Further clinical studies are therefore needed to determine the most effective dose and dosing schedule, evaluate the durability of vaccine-induced immunity and confirm long-term safety.

## 4. mRNA Vaccines Against Hantaviruses

### 4.1. Mechanism of Action of mRNA Vaccines

The mRNA vaccines are a flexible nucleic acid vaccine platform in which mRNA encoding a selected antigen is delivered into host cells and translated into the corresponding protein. Unlike DNA vaccines, mRNA vaccines do not require nuclear entry or transcription, as the delivered mRNA can directly engage the cytoplasmic translation machinery [78,79]. Following administration, mRNA is commonly encapsulated in lipid nanoparticles (LNPs), which protect the RNA from degradation and facilitate cellular uptake. LNPs are internalized predominantly through endocytosis, after which the mRNA must escape from the endosomal compartment and be released into the cytoplasm, where it can associate with ribosomes and undergo translation into the encoded antigen [80,81]. Thus, mRNA vaccines bypass the need for nuclear translocation and transcription, although efficient endosomal escape remains an important intracellular barrier to antigen expression [80,81]. By avoiding these additional intracellular barriers, mRNA vaccines can achieve antigen expression more directly and may therefore require lower doses than DNA vaccines to elicit an effective immune response [79].

Although nuclear entry, reverse transcription and genomic integration are not required for mRNA vaccine-mediated antigen expression, eukaryotic cells possess endogenous retrotransposition machinery that should be distinguished from the intended mechanism of LNP–mRNA vaccination. Eukaryotic genomes contain transposable elements (TEs), defined as mobile repetitive DNA sequences. These elements have played a fundamental role in shaping genome architecture and driving genomic plasticity throughout evolution. There are two groups of TEs: class I elements and class II elements. The first group is represented by retrotransposons, which mobilize through an RNA intermediate. The second group is composed of DNA transposons that mobilize via a DNA intermediate [82]. Among retrotransposons, a prominent example is the LINE-1 (L1) element, which encodes open reading frame 1 protein (ORF1p) and open reading frame 2 protein (ORF2p). ORF1p is a homotrimeric RNA-binding chaperone involved in nuclear entry. ORF1p with ORF2p and L1 RNA form a ribonucleoprotein (RNP) complex. ORF2p works as a multifunctional enzyme possessing an N-terminal apurinic/apyrimidinic endonuclease (APE-like EN) and a reverse transcriptase (RT) domain, which together drive target-primed reverse transcription (TPRT) and genomic integration [83]. While such retrotransposition processes represent an intrinsic biological mechanism of eukaryotic cells, they are completely distinct from how synthetic LNP–mRNA vaccines function. Vaccine mRNA does not encode reverse transcriptase or integrase. After escaping the endosome and entering the cytoplasm, delivered mRNA molecules are directly translated and subsequently degraded [80,84]. Thus, reverse transcription and genomic integration are not part of the intended mechanism of action of LNP–mRNA vaccines.

The resulting antigen stimulates both humoral and cellular immune responses, including antigen-specific B-cell and T-cell responses [78,79]. Nucleoside modifications, particularly N1-methylpseudouridine (m1Ψ), can improve mRNA translation and reduce unwanted innate immune activation, while lipid nanoparticles (LNPs) facilitate mRNA delivery into cells [78,79].

In hantavirus vaccine development, the M genomic segment, which encodes the glycoprotein precursor giving rise to the Gn and Gc glycoproteins, represents an important target for mRNA-based vaccine design [18,52,85]. These glycoproteins are expressed following vaccination and induce antigen-specific immune responses [18,52,85].

In a 2024 study, Zhang et al. [85] developed an mRNA vaccine encoding HTNV glycoproteins. The vaccine induced robust humoral and cellular immune responses and provided protective efficacy in mice, with a particularly strong virus-specific Th1 response [85]. Similarly, an mRNA-LNP vaccine encoding a prefusion-stabilized HTNV glycoprotein induced potent neutralizing antibody responses and protected mice against viral challenge [52]. This evidence supports the potential of mRNA vaccines as a strategy for the prevention of HTNV infection and demonstrates protection against viral challenge in the respective animal models. However, such preclinical protection should not be interpreted as evidence of clinical efficacy in humans.

### 4.2. Immunological Response Induced by mRNA Vaccines

Available preclinical evidence suggests that the protection induced by mRNA vaccines against hantaviruses may involve both humoral and cellular immune responses [78,79]. Following antigen expression, viral glycoproteins such as Gn and Gc are recognized by the immune system and stimulate antigen-specific B and T cells [78,79]. Activation of B cells and germinal-center formation promote the generation of high-affinity antibodies, including neutralizing antibodies capable of interfering with viral entry [52,79]. T-cell responses, particularly Th1 responses, may further contribute to viral clearance and the development of immunological memory [78,85]. In preclinical HTNV studies, mRNA vaccination induced both neutralizing antibody responses and strong Th1-associated cellular immunity, while prefusion-stabilized glycoprotein vaccination additionally enhanced germinal-center responses [52,85]. In ANDV models, mRNA vaccination similarly induced neutralizing antibodies and cellular immune responses associated with protection against viral challenge [86,87]. Together, these findings suggest that both humoral and cellular immune responses may contribute to protection against hantaviruses; however, their relative contributions and the extent to which either response represents a reliable correlate of protection remain uncertain [52,85,86,87].

### 4.3. Preclinical Studies of mRNA Vaccines Against Hantaviruses

Preclinical studies of mRNA vaccines against hantaviruses have so far focused mainly on HTNV and ANDV [52,85,86,87]. For HTNV, Zhang et al. [85] compared an mRNA vaccine encoding HTNV glycoproteins with other nucleic acid vaccine candidates in mice. The mRNA vaccine induced robust and sustained humoral and cellular immune responses and showed protective efficacy comparable to that of an inactivated vaccine [85]. Notably, it induced a particularly strong HTNV-specific Th1 response, whereas higher neutralizing antibody levels were observed with the DNA-LNP formulation [85].

Further optimization of the HTNV vaccine focused on antigen design. Ye et al. developed a prefusion-stabilized HTNV glycoprotein (GP-C3) and incorporated it into an mRNA-LNP vaccine [52]. The vaccine induced potent neutralizing antibody responses, enhanced germinal-center formation and protected mice against high-dose HTNV challenge [52].

For ANDV, Kuzmin et al. compared conventional uridine-containing mRNA (U-mRNA) with N1-methylpseudouridine-modified mRNA (m1Ψ-mRNA), both encoding the viral Gn/Gc glycoprotein precursor [86]. The study evaluated glycoprotein expression, innate immune activation, antibody responses, germinal-center formation and protective efficacy [86]. Both formulations induced neutralizing antibodies and protected Syrian hamsters against lethal ANDV challenge at the higher dose, while m1Ψ-mRNA showed reduced innate immune activation [86].

More recently, Meyer et al. investigated a single-dose ANDV mRNA vaccine and demonstrated protection from overt disease in golden Syrian hamsters, including at the lowest tested dose of 1 μg [87]. These results suggest that mRNA vaccines may provide effective protection against hantaviruses while allowing for further optimization of antigen design, nucleoside modification and vaccination schedules [52,85,86,87].

Overall, the available evidence remains limited to preclinical studies, mainly involving HTNV and ANDV [52,85,86,87]. Comparable primary mRNA vaccine studies against PUUV have not been identified. A key observation is that no clinical trials evaluating mRNA vaccines against HTNV, PUUV, or ANDV were identified in ClinicalTrials.gov. Therefore, the safety, optimal dosing, durability of protection and efficacy of these vaccines in humans remain unknown.

## 5. Limitations of DNA and mRNA Vaccines

A major limitation of DNA vaccines is the complex intracellular trafficking required before antigen expression can begin. Following cellular uptake, plasmid DNA must cross the plasma membrane and reach the nucleus, where it is transcribed by the host transcriptional machinery. The resulting mRNA transcripts must then be exported through the nuclear pore complex into the cytoplasm, where ribosomal translation generates the encoded antigen [88]. This additional requirement imposes substantial intracellular delivery constraints and constitutes an important mechanistic determinant of the differences in dose requirements and expression efficiency observed between DNA- and mRNA-based vaccine platforms [89]. These mechanistic differences may partly explain why mRNA platforms can achieve robust antigen expression at lower nucleic acid doses, whereas DNA vaccines often require optimization of delivery systems or physical delivery approaches to enhance cellular uptake and nuclear availability.

The obligatory nuclear localization of plasmid DNA has also prompted theoretical concerns regarding the possibility of integration into the host genome, potentially disrupting endogenous genes, altering regulatory regions, activating proto-oncogenic pathways, or contributing to genomic instability [89,90,91]. However, available preclinical investigations indicate that the frequency of plasmid integration is extremely low and remains below the estimated background frequency of spontaneous genomic alterations [88,89,92].

Repeated administration of nucleic acid vaccines (both DNA and mRNA) may raise safety concerns related to anti-nucleic acid immune responses and the potential induction of systemic autoimmune disorders [93,94,95]. Clinical evidence remains limited; however, a phase 1 trial involving 66 recipients of a cancer DNA vaccine detected neither anti-double-stranded DNA antibodies nor plasmid DNA in PBMCs, and no autoimmune manifestations were reported [96]. Larger clinical studies are still required to confirm these findings.

mRNA vaccines also present several limitations, including hypersensitivity reactions. They may be associated with components of lipid-based delivery systems [93,94,95]. In preclinical studies on COVID-19 vaccines, some LNP formulations, under specific experimental conditions, were associated with high mortality in animals [97], and showed marked inflammatory effects [98,99]. This issue has not yet been specifically investigated in hantavirus vaccine studies. Preliminary studies of HTNV mRNA vaccines reported no local adverse reactions or significant histopathological changes in major organs (gastrocnemius muscle, heart, liver, kidney and spleen) [85], while a more recent mRNA-LNP vaccine candidate for HTNV showed no signs of systemic toxicity in immunized mice [52].

The intrinsic instability of mRNA and its susceptibility to degradation create challenges related to formulation, storage and delivery [84,100]. Lipid nanoparticles can improve mRNA stability and intracellular delivery, but their formulation and physicochemical properties may influence the stability and performance of mRNA vaccines [100,101]. In the context of hantavirus vaccination, an important limitation is the limited preclinical evidence and the absence of advanced clinical evaluation of mRNA-based candidates. Nevertheless, studies in HTNV models have demonstrated promising immunogenicity and protective efficacy, supporting further investigation of this platform [52,85].

## 6. Materials and Methods

A comprehensive literature search was conducted using PubMed, Scopus and Google Scholar, focusing primarily on studies published within the past 10 years (2016–2026), while earlier pivotal publications were also included when considered relevant to the development of hantavirus vaccines. The data were searched on 4 August 2026 using terms: “Puumala” AND “vaccine”, “Hantaan” AND “vaccine”, “Andes” AND “vaccine”, as well as the corresponding abbreviations: “PUUV” AND “vaccine”, “HTNV” AND “vaccine”, and “ANDV” AND “vaccine”. ClinicalTrials.gov was searched using the same terms. Inclusion criteria were: (1) peer-reviewed original research articles reporting preclinical or clinical studies of DNA- or mRNA-based hantavirus vaccines; (2) systematic and narrative reviews published in peer-reviewed journals and relevant to hantavirus vaccine development, antigen design, immunogenicity, safety or delivery strategies; and (3) articles published in English. Exclusion criteria were: (1) non-peer-reviewed reports and other publications lacking formal peer review; (2) conference abstracts without full-text availability; (3) studies focusing exclusively on conventional vaccine platforms without relevance to nucleic acid vaccine development; and (4) publications of insufficient scientific quality or relevance to the scope of this review. All identified relevant hantavirus vaccine trials are summarized in Table 2.

Figure 1 was created using GoodNotes 6 for iPad (v.6.3.55, Goodnotes Inc., London, UK)—all based on the authors’ interpretation of synthesized data from multiple studies. The graphical abstract was prepared using GoodNotes 6 for iPad (v.6.3.55, Goodnotes Inc., London, UK) and Canva Premium (Canva Pty Ltd., Sydney, Australia).

To provide a quantitative synthesis of the available clinical evidence, data on safety and immunogenicity were extracted from the included studies as the number of participants experiencing a given outcome and the corresponding number of evaluable participants (*n*/*n*), together with the percentage reported in the original publication. Where the underlying numerator and denominator were available, 95% confidence intervals (CIs) for proportions were calculated using the exact Clopper–Pearson method. *p*-values were retained only when direct statistical comparisons between groups were reported in the original clinical study. Because the included studies differed substantially with respect to vaccine construct, viral target, dose, delivery system, vaccination schedule, study population, sampling time points, immunological assays and definitions of seropositivity or seroconversion, the reported outcome proportions were not pooled across studies. Instead, quantitative results were presented at the individual-study or cohort level to preserve clinically relevant differences between studies and to avoid inappropriate aggregation of heterogeneous data. Where the original publication did not provide sufficient information to determine the exact numerator and denominator, a confidence interval was not calculated.

## 7. Discussion, Conclusions and Future Directions

Hantaviruses remain an important public health concern due to their broad geographic distribution, zoonotic nature and potential to cause severe clinical syndromes, including HFRS and HCPS. Although inactivated vaccines are available and have been used in endemic regions of Asia, no broadly available, internationally licensed vaccine against hantavirus infection currently exists. The development of nucleic acid vaccine platforms, particularly DNA and mRNA vaccines, has therefore emerged as a promising approach to address the limitations associated with conventional hantavirus vaccine strategies. However, the translational maturity of these two platforms differs substantially. DNA vaccines against hantaviruses have progressed into early-phase clinical evaluation, providing initial human safety and immunogenicity data, whereas mRNA-based candidates remain predominantly at the preclinical stage, with evidence currently limited to experimental models. Therefore, although both platforms are promising, their current levels of clinical validation should not be considered equivalent.

Preclinical studies have provided substantial evidence that nucleic acid vaccines can induce both humoral and cellular immune responses against pathogenic *orthohantaviruses*. Among the antigens evaluated, the viral envelope glycoproteins Gn and Gc encoded by the M segment represent the principal targets because of their critical role in viral entry and their accessibility to neutralizing antibodies. Early DNA vaccine studies demonstrated that glycoprotein-based vaccination can induce neutralizing antibodies and protect experimental animals against homologous viral challenge. Studies involving HTNV, SEOV, DOBV, PUUV and ANDV have also demonstrated varying degrees of cross-reactive and cross-protective immunity, although the breadth of protection is strongly influenced by the antigenic relatedness of the viruses. These results highlight both the potential and the limitations of developing broadly protective hantavirus vaccines based on a single viral antigen.

Advances in bioinformatics and computational biology have markedly accelerated progress in this field [101,102,103]. Comprehensive computational analyses of *orthohantavirus* motifs and codon usage have yielded important insights into host immune regulation and viral evolutionary history, facilitating the identification of conserved epitopes and antigenic regions essential for eliciting robust immune responses [104,105]. In addition, molecular docking and dynamic simulation approaches are employed to assess the binding affinity between vaccine candidate proteins and host immune receptors [106], while immune simulation analyses enable the prediction of natural immune responses [107]. Several multi-epitope subunit vaccines designed to provide cross-protection against *orthohantaviruses*, including HTNV and PUUV, have been developed using immunoinformatics-based strategies [101,102,106,107,108]. Despite this evidence, all computationally designed vaccines require thorough experimental validation prior to clinical application.

Recent advances in antigen design have further expanded the potential of nucleic acid vaccines against hantaviruses. Structural studies have demonstrated that the Gn/Gc glycoprotein complex adopts a metastable prefusion conformation and that neutralizing antibodies can recognize conformational and quaternary epitopes formed within Gc or at the Gn/Gc interface [49,50,51,52,53]. These data indicate that effective antigen design should preserve the relevant structural and antigenic properties of the viral glycoproteins rather than focus exclusively on optimizing nucleotide sequences or plasmid expression.

A novel HTNV study provides direct experimental support for this concept. A prefusion-stabilized glycoprotein antigen generated by structure-guided engineering induced stronger neutralizing antibody responses and enhanced germinal-center activity compared with non-stabilized constructs. Notably, the optimized antigen was effective when delivered using both DNA and mRNA-LNP platforms, demonstrating that structural antigen engineering can complement different nucleic acid vaccine technologies [52]. Structural characterization of HTNV Gn and broadly neutralizing hantavirus antibodies further indicates that rational epitope selection should take into account epitope accessibility, conformational integrity and conservation of structurally relevant antigenic regions [49,50,51,52,53]. Taken together, the available evidence indicates that an integrated approach combining nucleotide-level optimization, structure-guided protein engineering and rational epitope selection in the development of next-generation hantavirus vaccines.

The clinical development of DNA vaccines has provided an important translational extension of these preclinical findings. Early-phase studies targeting HTNV, PUUV and ANDV have demonstrated that DNA vaccination can induce measurable neutralizing-antibody responses and has generally shown an acceptable safety and tolerability profile. However, the magnitude and consistency of immunogenicity have varied between studies, vaccine formulations and vaccination schedules. In particular, studies of combined HTNV and PUUV vaccines indicate that simultaneous targeting of multiple hantaviruses does not necessarily result in equivalent immune responses against each component, emphasizing the potential challenge of antigenic or immunological interference in multivalent formulations. Moreover, differences in neutralization assays, response definitions, dosing schedules and administration strategies complicate direct comparisons across clinical studies.

mRNA vaccine platforms offer an additional opportunity to overcome some limitations associated with conventional DNA vaccination. Their transient expression profile and flexibility in antigen design allow for rapid adaptation to newly characterized viral strains and facilitate the incorporation of structurally optimized or multivalent antigens. Together with recent advances in lipid nanoparticle-based formulations and structure-guided antigen engineering, these characteristics make mRNA technology particularly attractive for the development of next-generation hantavirus vaccines. Nevertheless, the current evidence base for mRNA vaccines remains less mature than that for DNA vaccines, and further preclinical studies are required to establish the durability, breadth and protective efficacy of mRNA-induced immunity against diverse pathogenic *orthohantaviruses*.

Several major challenges therefore remain before nucleic acid vaccines can be translated into broadly protective hantavirus vaccines. These include the considerable antigenic diversity among hantaviruses, incomplete cross-protection between Old World and New World viruses, uncertainty regarding correlates of protection, the absence of an animal model that fully reproduces human disease and the need to establish durable immunity. The development of multivalent or pan-hantavirus vaccine strategies will likely require careful selection and structural optimization of conserved antigenic regions while maintaining the ability to induce potent neutralizing and cellular responses. In addition, standardized preclinical and clinical evaluation criteria would facilitate comparison between vaccine candidates and improve the interpretation of immunogenicity data.

The accumulated evidence supports DNA and mRNA vaccination as promising platforms for hantavirus prevention. The progression from early proof-of-concept DNA vaccines to structure-guided and increasingly sophisticated nucleic acid vaccine candidates demonstrates substantial progress in the field. Future research should focus on defining the immunological correlates of protection, improving the breadth and durability of vaccine-induced immunity and developing multivalent or broadly protective formulations capable of targeting the major pathogenic *orthohantaviruses*. Continued integration of structural virology, immunology, reverse vaccinology and nucleic acid vaccine technology may ultimately enable the development of a safe, durable and broadly effective hantavirus vaccine.

## Figures and Tables

**Figure 1:**
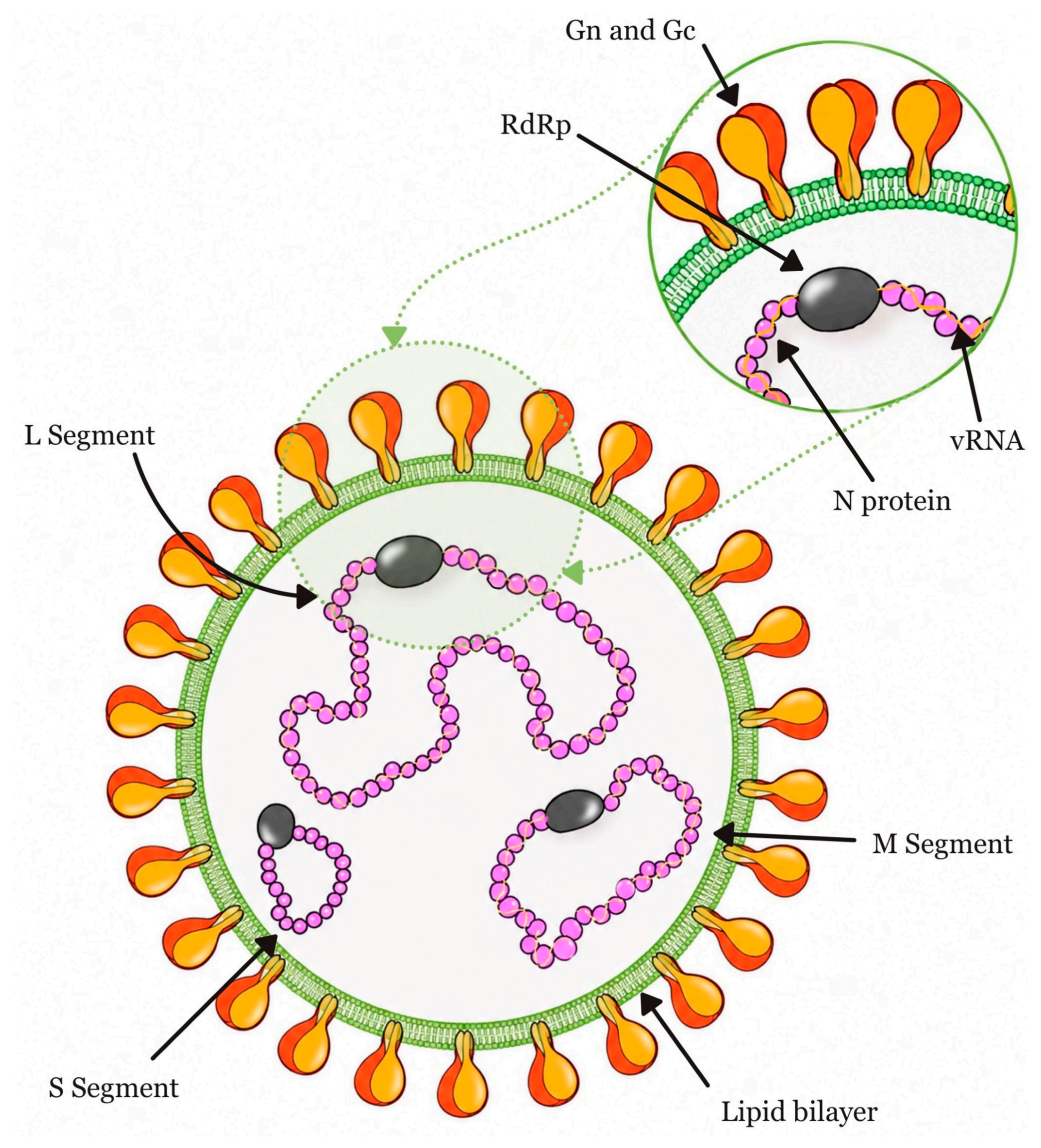
Schematic structure of hantaviruses [21,22]. The virion consists of a host-derived lipid bilayer containing surface glycoproteins Gn and Gc. The genome comprises three negative-sense RNA segments: the L segment, M segment and S segment. Each vRNA segment is associated with the N protein and the viral RNA-dependent RNA polymerase (RdRp), forming ribonucleoprotein complexes enclosed within the viral envelope.

**Table 1:** Hantavirus genome segments, encoded proteins and their functional roles in the viral replication cycle.

Segment	Encoded Viral Protein(s)	Main Function of Protein	References
S	Nucleoprotein/Nucleocapsid protein (N)	Encapsidates viral genomic RNA and forms vRNP complexes associated with the L protein; supports genome packaging, replication and transcription.	[28,29]
	Nonstructural protein (NSs)	NSs may act as an antagonist of type I interferon induction and contribute to viral immune evasion (this function has been shown for ANDV NSs and for PUUV NSs).	[30,31,32]
M	Glycoprotein precursor GPC → Gn and Gc glycoproteins	Gc and Gn form the spike complex responsible for receptor-binding. Gc acts as a class II fusion protein mediating endosomal membrane fusion. G_N_ binds to N and RNA, supporting recruitment of vRNPs to sites of assembly and genome packaging into virions. They are the principal surface-exposed antigens and major targets of neutralizing antibodies.	[33,34,35,36]
L	L protein, which contains the RdRp and endonuclease	Catalyzes viral genome replication and transcription within vRNP complexes composed of genomic RNA, N protein and L protein. Mediates cap-snatching to acquire 5′ capped host-derived primers for viral mRNA transcription.	[37]

Abbreviations: ANDV—Andes virus; GPC—glycoprotein precursor; Gn—glycoprotein N; Gc—glycoprotein C; N—nucleocapsid protein; NSs—nonstructural protein; PUUV—Puumala virus; RdRp—RNA-dependent RNA polymerase; RNA—ribonucleic acid; vRNP—viral ribonucleoprotein complex.

## Data Availability

No new data were created or analyzed in this study. Data sharing is not applicable to this article.

## Abbreviations

The following abbreviations are used in this manuscript:

ANDV	Andes virus
GPC	Glycoprotein precursor
Gn	Glycoprotein N
Gc	Glycoprotein C
N	Nucleocapsid protein
NSs	Nonstructural protein
PUUV	Puumala virus
RdRp	RNA-dependent RNA polymerase
RNA	Ribonucleic acid
vRNP	Viral ribonucleoprotein complex
HTNV	Hantaan virus
DNA	Deoxyribonucleic acid
IM	Intramuscular
ID	Intradermal
U.S.	United States
mg	milligram
AE	Adverse event
SAEs	Serious adverse events
PRNT_50_	50% plaque reduction neutralization test
PsVNA_50_	50% pseudovirion neutralization assay
